# Neural stem cell mediated recovery is enhanced by Chondroitinase ABC pretreatment in chronic cervical spinal cord injury

**DOI:** 10.1371/journal.pone.0182339

**Published:** 2017-08-03

**Authors:** Hidenori Suzuki, Christopher S. Ahuja, Ryan P. Salewski, Lijun Li, Kajana Satkunendrarajah, Narihito Nagoshi, Shinsuke Shibata, Michael G. Fehlings

**Affiliations:** 1 Division of Genetics and Development, Krembil Research Institute, Toronto, Canada; 2 Department of Orthopedics Surgery, Yamaguchi University Graduate School of Medicine, Ube, Japan; 3 Institute of Medical Science, University of Toronto, Toronto, Canada; 4 Division of Neurosurgery, University of Toronto, University of Toronto, Toronto, Canada; 5 Faculty of Medicine, University of Toronto, Toronto, Canada; 6 Department of Orthopedics Surgery, Keio University, Tokyo, Japan; 7 Department of Physiology, Keio University, Tokyo, Japan; 8 Spinal Program, University Health Network, Toronto Western Hospital, Toronto, Canada; Lewis Katz School of Medicine at Temple University, UNITED STATES

## Abstract

Traumatic spinal cord injuries (SCIs) affect millions of people worldwide; the majority of whom are in the chronic phase of their injury. Unfortunately, most current treatments target the acute/subacute injury phase as the microenvironment of chronically injured cord consists of a well-established glial scar with inhibitory chondroitin sulfate proteoglycans (CSPGs) which acts as a potent barrier to regeneration. It has been shown that CSPGs can be degraded *in vivo* by intrathecal Chondroitinase ABC (ChABC) to produce a more permissive environment for regeneration by endogenous cells or transplanted neural stem cells (NSCs) in the subacute phase of injury. Using a translationally-relevant clip-contusion model of cervical spinal cord injury in mice we sought to determine if ChABC pretreatment could modify the harsh chronic microenvironment to enhance subsequent regeneration by induced pluripotent stem cell-derived NSCs (iPS-NSC). Seven weeks after injury—during the chronic phase—we delivered ChABC by intrathecal osmotic pump for one week followed by intraparenchymal iPS-NSC transplant rostral and caudal to the injury epicenter. ChABC administration reduced chronic-injury scar and resulted in significantly improved iPSC-NSC survival with clear differentiation into all three neuroglial lineages. Neurons derived from transplanted cells also formed functional synapses with host circuits on patch clamp analysis. Furthermore, the combined treatment led to recovery in key functional muscle groups including forelimb grip strength and measures of forelimb/hindlimb locomotion assessed by Catwalk. This represents important proof-of-concept data that the chronically injured spinal cord can be ‘unlocked’ by ChABC pretreatment to produce a microenvironment conducive to regenerative iPS-NSC therapy.

## Introduction

Traumatic spinal cord injury (SCI) interrupts sensory and motor tracts resulting in severe, lifelong functional impairments for patients. These injuries most often occur in the cervical spine leading to quadriplegia, which is particularly disabling due to the impact that loss of hand function has on a patient’s ability to perform basic self-care (e.g. feeding, hygiene, etc.)[1–3] Data from the United States estimates the lifetime cost of care for a 25 year-old who sustains a high cervical injury to be as high as $4.7 million USD[4, 5]. The vast majority of neuroregenerative interventions have focused on treating these patients in the acute or subacute periods when salvageable neuroglial cells may still exist and the glial scar has not yet been fully established[5–8]. Unfortunately, over 95% of patients with SCI are in the chronic phase of their injury[4].

Despite the need, one of the greatest challenges in developing an effective therapy for chronic SCI has been the inhibitory microenvironment of the injured spinal cord. After SCI, astrocytes activate, proliferate, and migrate to the perilesional region where they form a dense interwoven network of processes and deposit chondroitin sulfate proteoglycans (CSPG) into the extracellular matrix [9]. Dystrophic axons surround the injury epicenter becoming trapped in this dense meshwork of scar tissue[9, 10]. The axonal failure is mediated by both the physical proteoglycan barrier and biochemical signaling within axon growth cones induced by interaction with sulfated CSPGs[11–13].

Chondroitinase ABC (ChABC) is a bacterially-derived enzyme capable of degrading CSPGs and has been successfully used as a standalone treatment in animal models of SCI. Experiments performed in a dorsal column crush model were among the first to show that intrathecal delivery of ChABC promotes axonal sprouting and regrowth in both ascending and descending tracts[14, 15]. A subsequent study demonstrated that intrathecal delivery of ChABC was effective at improving locomotor and bladder function in a contusion model of thoracic injury [16].

However, the regenerative capacity of local cells may be limited which has encouraged the development of cell transplantation strategies. Exogenous neural stem cell (NSC) therapies are particularly promising as the cells have the potential to differentiate into all three neuroglial lineages (e.g. neurons, astrocytes, and oligodendrocytes) to regenerate neural circuits, remyelinate denuded axons, and provide trophic support to endogenous cells [8, 17–19]. Previous work by our group has also shown that ChABC can be successfully combined with subacute cell transplantation to improve the survival and integration of adult-derived NSCs in a rat model of contusion-compression thoracic SCI [20].

In the present study, we build on this work by assessing a novel combinatorial treatment strategy in chronic SCI. Our approach is to use ChABC pretreatment to positively modify the injured cord microenvironment prior to transplantation of translationally-relevant, non-virally generated, induced pluripotent stem cell derived NSCs (iPS-NSC). For the purposes of this study we used a mouse model of cervical clip-compression injury developed in our laboratory that is similar to our well-established rat model[21–23]. This method creates a bilateral, incomplete injury that very closely mimics the pathophysiology of human SCI. Our overall goal is to improve iPS-NSC survival and integration with ChABC pretreatment in chronic cervical SCI to enhance forelimb recovery.

## Experimental procedures

### Animals

**Fig 1:**
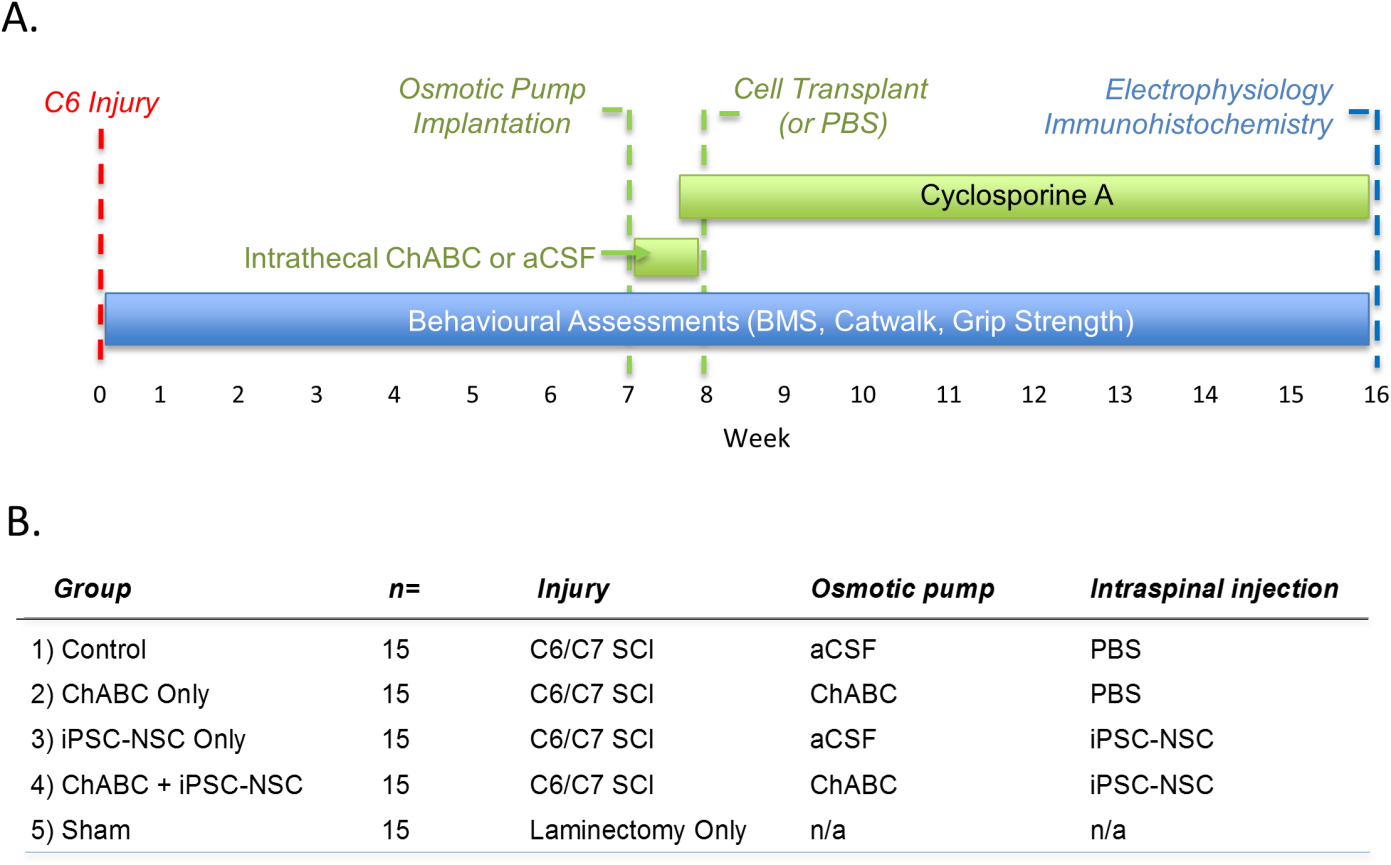
Schematic representation of experimental design. Treatments are in green; assessments are in blue. (A) A C6 clip-contusion spinal cord injury (SCI) was performed at 0 weeks in C57/BL6 mice. Seven weeks post-injury, a mini-osmotic pump was implanted rostral to the injury site. The pump delivered either Chondroitinase ABC (ChABC) in artificial cerebral spinal fluid (aCSF) or aCSF alone. One week later the pump was explanted and induced pluripotent stem cell-derived neural stem cells (iPS-NSC) were transplanted into the cord parenchyma at two rostral and two caudal sites. 50,000 cells in 1μL were delivered to each site. Cyclosporine A immunosuppression was delivered from 2 days prior to transplantation until the end of the study. Behavioral assessments were performed across all 16 weeks of the study. At 16 weeks electrophysiologic assessments were completed followed by whole-animal perfusion, fixation and immunohistochemical analyses. (B) Outline of the control and treatment groups. For additional details please see S1 Table.

All experimental protocols were approved by the Animal Care Committee at Krembil Research Institute (Toronto, Canada). Guidelines from the Canadian Council on Animal Care were strictly followed. All animals received food and water *ad libitum* throughout the study. A total of 70 adult (8–10 weeks old), female, wild-type C57BL/6 mice (15 to 20g) from Jackson Laboratories (Bar Harbor, ME, USA) were the subjects of this study. All surgeries were performed under deep anesthesia with isofluorane (2% induction; 1% maintenance) in a 1:1 mixture of oxygen and nitrous oxide. Ten mice received a C6/7 laminectomy alone (sham control group). Sixty mice received a C6/7 clip-contusion injury as described below. Six weeks after injury, mice were randomized into four experimental groups (control, ChABC, iPS-NSCs, & ChABC + iPS-NSCs; Fig 1). Grip strength measurements at six weeks were not statistically different between groups (ANOVA; p = 0.871).

### iPS-NSC generation

Mouse embryonic fibroblasts (MEF) for use as an iPS feeder layer were prepared from CD1 embryos following a protocol described by Nagy et al.[24] iPS cells were generated from CD1 mouse fibroblasts using the piggyBac (PB) transposon system reported by Woltjen et al.[25] In brief, fibroblasts were plated on gelatinized 6-well plates at 7.5x10^4^ cells/well. After 24 hours they were transfected using FugeneHD (Roche, Basel, Switzerland) with 2μg/well of a DNA mixture containing expression vectors for the PB transposase and PB transposons. These contained a CAG promoter driven rtTA, a CAG-GFP, and a set of tetracycline inducible promoter (Tet-On) driven reprogramming factors (polycistronic cMyc, Klf4, Oct4 and Sox2) linked by self-cleaving 2A sequences. 24hrs after transfection, the medium was supplemented with 1.5μg/ml doxycycline and 48hrs after transfection the cells were transitioned to mouse ESC medium containing 1,000U/mL leukemia inhibitory factor (LIF; Sigma-Aldrich, St Louis, MO) and 1.5μg/ml doxycycline. On day 10–14, colonies morphologically resembling ESCs were picked and expanded on mitomycin-c arrested MEF feeders. Lines typically became doxycycline-independent 21 days after transfection.

Definitive NSCs were generated using a well-established protocol previously described by our lab [18, 26]. Briefly, iPS colonies were picked and dissociated to single cells using TrypLE. Cells were seeded at 10 cells/μL in low-attachment culture flasks contain serum-free media (SFM) with LIF to yield primitive neurospheres after 7 days. Definitive neurospheres containing iPS-NSC were generated by dissociating and expanding primitive neurospheres in SFM containing 10μg/mL fibroblast growth factor (FGF; Sigma-Aldrich), 2μg/mL heparin (Sigma-Aldrich), B-27 supplement (Gibco, Grand Island, NY) and Delta-like 4 Notch ligand (Dll4; R&D Systems Inc., Minneapolis, MN).

### Spinal cord injury

Mice were anesthetized with inhaled isoflurane (2% induction; 1% maintenance) in a 1:1 mixture of nitrous oxide and oxygen. Using strict aseptic technique, a C6 and C7 laminectomy was completed using a dorsal midline approach. The dura was gently dissected circumferentially to allow the ventral blade of a modified aneurysm clip (FEJOTA mouse clip, University Health Network, Canada) to be passed at C6/7 and closed on the cord as described in our previous reports (S1 Fig)[18]. A closing force of 6.5g for 40 seconds was used. Sham injured mice received a laminectomy only without dural dissection or clip injury. 1mL Normal Saline and 0.05mg/kg buprenorphine were administered subcutaneously immediately after the operation. Animals were recovered in sterile cages under heating lamps and then housed in a temperature-controlled room (27°C) for the length of the study. Food and water were provided ad libitum. Water was supplemented with Clavamox antibiotic (60mg/L water; Pfizer Animal Health, New York, NY) from 2 days pre-operatively to 3 days post-operatively. Buprenorphine was administered twice daily for 2 days after surgery. The animals’ bladders were manually expressed twice daily until the return of spontaneous bladder function.

### Chondroitinase ABC injection

7 weeks after injury and 1 week prior to cell transplantation mice were randomly assigned to receive either artificial cerebrospinal fluid (aCSF) or aCSF with ChABC (3.64mU/mL; amsbio, Abingdon, UK) (S1 Table). Under isofluorane anesthetic (as above) the injured spinal cord was surgically exposed and a fine (0.23 mm OD; 0.09 mm ID) intrathecal catheter (Mouse IT catheter 0007743; Alzet, Cupertino, CA; S1D Fig) was implanted and connected to an osmotic mini-pump (Model 1007D, Alzet) as described by Karimi-Abdolrezaee et al.[20] The treatment fluid was delivered at 0.5μl/hr for 7 days followed by surgical explantation at the time of cell transplant.

### iPS-NSC transplantation

iPS-NSC transplantations were performed as described by Salewski et al.[18] Briefly, under isofluorane anesthetic the injured spinal cord was exposed and the intrathecal catheter and osmotic pump were removed. Mice were randomly assigned to receive iPS-NSC or cell-free PBS (control; S1 Table). Four injection sites were mapped bilaterally adjacent to the midline dorsal vein at 1mm rostral and 1mm caudal to the injury epicenter. The dura at each site was punctured using a fine needle. A pulled glass micropipette attached to a 5μL Hamilton syringe on a computer-controlled syringe pump (World Precision Instruments) was used to stereotactically transplant 1μL (at 0.2μl/min) of PBS alone or 50,000 cells in PBS into the underlying spinal cord at each of the four sites. Immunosuppression with subcutaneous Cyclosporine A (20mg/kg daily) was started 2 days prior to transplantation and continued until animal sacrifice.

### Behavioral testing

All neurobehavioral assessments (N = 70) were performed and analyzed by two independent examiners blinded to the treatment groups. Functional recovery was assessed using the Basso Mouse Scale (BMS), CatWalk digital gait analysis system, forelimb grip strength meter and inclined plane test.

The BMS is a global measure of hindlimb function in mice [27] scored from 0 (no function) to 9 (normal movement, coordination, and stability). Mice were placed in an open field and evaluated once per week at the same day and time for 16 weeks by each observer.

The CatWalk gait analysis digitally records animals’ locomotion in a dark environment across a guided linear path. Key measures of coordinated 4-limb gait can be analyzed including swing speed and stride length [28]. We have previously demonstrated the utility of this tool in SCI models [29–31]. Mice were assessed at baseline and every two weeks from week 6 to 16 post-injury. Mice unable to traverse the catwalk platform were not included in the analysis.

Bilateral forelimb motor function was evaluated using a grip strength meter [29, 32] where animals held a grip bar and were rapidly pulled away to assess peak grip force. Seven consecutive trials were performed weekly for 16 weeks with the highest and lowest values trimmed to calculate a mean value at each time point.

### Perfusion and sectioning

Eight weeks after transplantation, animals were deeply anesthetized with 5% inhaled isoflurane and transcardially perfused with ice cold 0.1M PBS followed by 4% paraformaldehyde (PFA) in 0.1 M PBS (pH 7.4). A 2cm section of cervical cord centered on the lesion was harvested and post-fixed in 4% PFA overnight at 4°C followed by 48hrs of cryoprotection in 30% sucrose in PBS at 4°C. The sample was then snap frozen in M1 Embedding Matrix (Thermo Fisher Scientific, Waltham, MA) on dry ice. Serial 35μm thick cryosections were then arranged on Superfrost glass slides (Thermo Fisher Scientific) as either six cross-sectional slices or two longitudinal slices.

### Immunohistochemistry

Slides were blocked and permeabilized for 1 hour with PBS containing 1% bovine serum albumin (BSA), 5% normal goat serum (NGS), 5% nonfat milk powder and 0.25% Triton X-100. After 3 PBS washes, primary antibodies (S2 and S3 Tables) in the same blocking solution (without Triton X-100) were added to the slides overnight at 4°C. After 3 additional PBS washes, secondary antibody (Thermo Fisher Scientific) in blocking solution was added for 1 hour. After a final 3 PBS washes, coverslips were applied with Vectashield mounting medium (Vector Labs, Burlingame, CA) containing 4-6-diamidino-2-phenylindole (DAPI; Vector Labs) as a nuclear counterstain.

GFP^+^ cell counts (N = 12) were estimated using unbiased stereological techniques with Stereo Investigator’s optical fractionator software on a Nikon Eclipse E800 microscope. Random counting frames were evaluated on sections every 360μm from 2880μm rostral to 2880μm caudal to the injury epicenter (5760μm total). Differentiation of GFP^+^ cells (N = 10) was assessed using a Zeiss LSM-510 Meta Confocal microscope with data presented as the proportion of Nestin^+^/GFP^+^, NeuN^+^/GFP^+^, APC^+^/GFP^+^, or GFAP^+^/GFP^+^ cells among all imaged GFP^+^ cells.

Additional qualitative immunostains were completed to assess CSPGs (CS56^+^), myelin (MBP^+^), mature neurons (NF200^+^, MAP2^+^) and synapse formation (Synaptophysin^+^).

For immunointensity measurements of CSPG (CS56+) and GFAP, we selected and imaged three longitudinal / horizontal sections per mouse at 0 ± 300 μm ventral and dorsal to the epicenter. Each image was acquired using identical exposure time, gain and offset values. Using NIH ImageJ software, we traced the entire cord while excluding the cavity region. Integrated density and area measurements were calculated. Immunointensity was calculated by dividing integrated density by area. This calculation is useful to account for variability between animals in cord size.

### Quantification of motor neurons

Using the optical fractionator on a Nikon Eclipse E800 microscope, ChAT^+^/NeuN^+^ cells were counted on slices every 360μm from 1800μm rostral to 1800μm caudal to the lesion epicenter (3600μm total). A counting frame of 100x100μm was used. The total number of ChAT^+^/NeuN^+^ cells was estimated from this for 2 animals in the sham group and 4 animals in each experimental group (N = 18).

### Immuno-transmission electron microscopy

Spinal cords from the iPS-NSC and ChABC + iPS-NSC groups were fixed with 4% PFA in 0.1M PBS for 12 hours followed by incubation with 15% and 30% sucrose for 24 hours each. The tissue was embedded in cryoprotective compound, frozen, and sectioned at 20 μm thick using a Leica CM3050s cryostat onto Superfrost (FisherScientific) glass slides. Sections were then incubated with 5% Block Ace (DS Pharma Biomedical) with 0.01% Saponin in 0.1M PB for one hour and stained with primary rabbit anti-GFP antibody (1:100; MBL International) for 72 hours at 4°C followed by incubation with nanogold conjugated goat anti-rabbit secondary antibody (1:100; Invitrogen) for 24 hours at 4°C. After 2.5% glutaraldehyde fixation, nanogold signal was enhanced with R-Gent SE-EM Silver Enhancement Reagent (Aurion) for 30 minutes. Sections were fixed with 1.0% OsO4 for 90 minutes at 25°C, dehydrated through graded series of ethanol and embedded in Epon. Ultrathin sections (70 nm) were prepared with an ultramicrotome (Leica UC7) and double contrasted with uranyl acetate and lead citrate. The sections were imaged using a transmission EM (JEOL model 1400 plus).

### *In vivo* electrophysiology

The integrity and function of forelimb motor tracts were assessed using motor evoked potential (MEP) recordings. At 8 weeks after transplantation, mice were anesthetized with 2% inhaled isofluorane and the interlaminar ligaments between C1 and C2 were surgically removed. Two 1mm ball electrodes were positioned extradurally over the spinal cord at C1. Two stainless steel needle electrodes were inserted into the forelimb muscles. A constant current stimulus of 0.04ms duration and 2.0mA intensity was applied at a frequency of 0.13 Hz. Using a bandwidth of 10–3000Hz, a total of 50 MEPs were averaged and replicated. MEP peak latency was measured from the start of the stimulus (S) to the peak of the first negative peak (N1). The evoked potential amplitudes were measured as the voltage difference from the peak of the first negative peak (N1) to the peak of the second positive peak (P2).

### *Ex vivo* electrophysiology

Animals were deeply anesthetized with intraperitoneal sodium pentobarbital (60 mg/kg) and transcardially perfused with ice cold 95% O_2_ + 5% CO_2_ saturated high-sucrose artificial cerebrospinal fluid (aCSF) containing: 210mM sucrose, 26 mM NaHCO_3,_ 2.5mM KCl, 1mM CaCl_2,_ 4mM MgCl_2,_ 1.25mM NaH_2_PO_4_ and 10mM D-glucose. A full-length cord was quickly excised and submerged in the same ice cold saturated aCSF solution. The meninges were carefully removed and the cord was trimmed from cervicomedullary junction to upper thoracic cord. Sections were adhered to a 4% Agar block with cyanoacrylate glue and cut into six 120μm thick coronal sections on a Leica VT 1200s vibratome before being incubated for 1 hour at room temperature in another 95% O_2_-5% CO_2_ aCSF solution containing: 125mM NaCl; 26mM NaHCO_3_; 2.5mM KCl; 2mM CaCl_2_; 1.3mM MgSO_4_; 1.25mM NaH_2_PO_4_; 10mM D-glucose.

All recordings were performed on the most dorsal three slices under a continuous 1.5mL/min flow of oxygenated aCSF. Slices were visualized under infrared differential interference contrast (IR-DIC) using a Nikon E600FN microscope and Hamamatsu C2400 CCD camera. Patch clamp pipettes were pulled using an upright electrode puller (PP-83; Narishige, Japan) and polished using a micro-forge (MF-900; Narishige, Japan). The pipette resistance was 3–5 MΩ when filled with a solution (pH 7.3; 290-300mOsm) containing: 140mM CsCl, 2mM MgCl_2_, 1mM CaCl_2_, 5mM EGTA, 10mM HEPES and 4mM MgATP. Whole-cell patch clamp recording was performed on GFP^+^ cells and acquired with a multiClamp 700A amplifier using pClamp 10 software interfaced to a Digidata 1322A acquisition board (Molecular Devices, CA, USA). Input was filtered at 10 kHz and digitized at 250 kHz.

### Statistical analysis

Statistical analyses were performed using SPSS version 21 (IBM SPSS Statistics, Chicago, IL). Behavioral scores were analyzed using a mixed ANOVA comparing groups over time. Analyses were split into baseline (week 0), post-injury (weeks 1 to 6) and post-treatment (weeks 8 to 16) periods. Histological and electrophysiological data was analyzed using one-way ANOVA. Where appropriate, multiple comparisons were controlled with a post hoc Tukey HSD test. If omnibus tests were significant, individual comparisons were made between the control group and experimental group of interest using t-tests with Bonferroni correction. Data are reported as mean ± SEM. An alpha level of 0.05 was used.

## Results

### ChABC pre-treatment enhances iPS-NSC survival

**Fig 2:**
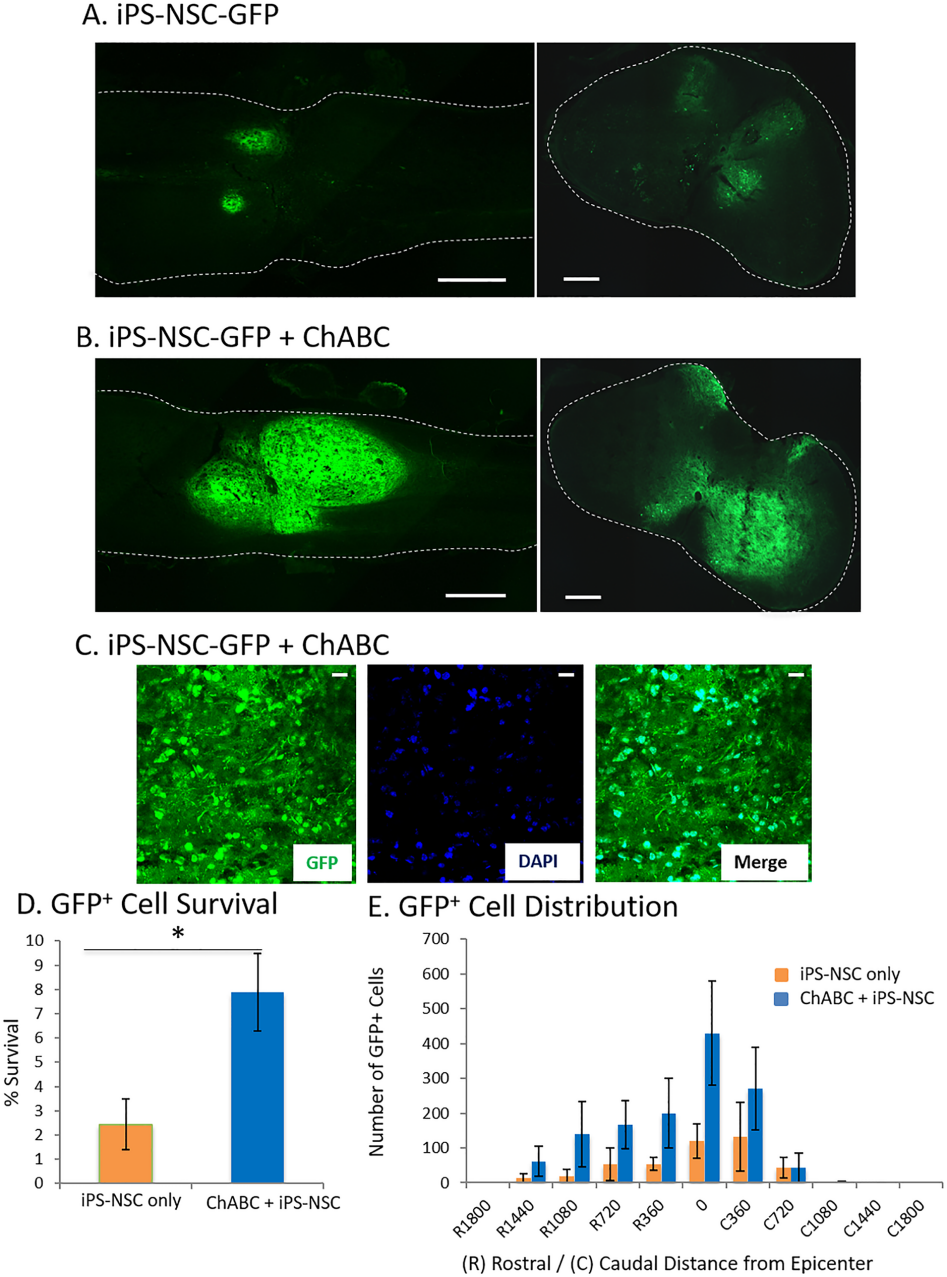
ChABC pretreatment enhanced transplanted iPSC-NSC survival. (A,B,C) Representative longitudinal and axial sections of injured spinal cords 8 weeks after transplant (16 weeks after injury). Scale bar represents 200 μm in A and B. Scale bar represents 50 μm in C. A greater number of GFP^+^ iPS-NSC can be seen throughout the ChABC pretreated cord than the control cord. (D) Quantification of GFP^+^ cell counts using optical fractionation and unbiased stereological techniques. GFP^+^ iPS-NSC survival was significantly higher in the ChABC pretreated group by more than 3 times. (E) Increased survival was seen in sections at the epicenter and rostrally/caudally up to 1440μm. Data represented as mean ± SEM; n = 6 per group. (*) indicates statistical significance at p<0.05

Pretreatment of chronically injured spinal cords with ChABC 1 week prior to transplant significantly increased the subsequent survival of iPS-NSC from 2.44 ± 1.04% (4876 ± 2079 cells) in the iPS-NSC group to 7.88 ± 1.60% (15769 ± 3195 cells) in the iPS-NSC + ChABC group at 8 weeks post-transplant (df = 10; t = 2.86; p = 0.017)(Fig 2A, 2B, 2C and 2D). Grafted iPS-NSC were not proliferating at 8 weeks post-transplant as no Ki67^+^/GFP^+^ cells could be found in either group (S2 Fig). There was no evidence of neoplastic cell growth in any of the transplanted animals in this study (data not shown).

The distribution of grafted cells strongly favored integration near the epicenter in both groups with 74.1 ± 7.4% of cells within 360μm of the epicenter in the iPS-NSC + ChABC group and 73.0 ± 11.0% in the iPS-NSC group despite injections at 1mm rostral and caudal to the epicenter (Fig 2E). There was no clear difference in ventrally/dorsally or left/right integration on cross-section in either group.

### iPS-NSC differentiation and integration *in vivo*

**Fig 3:**
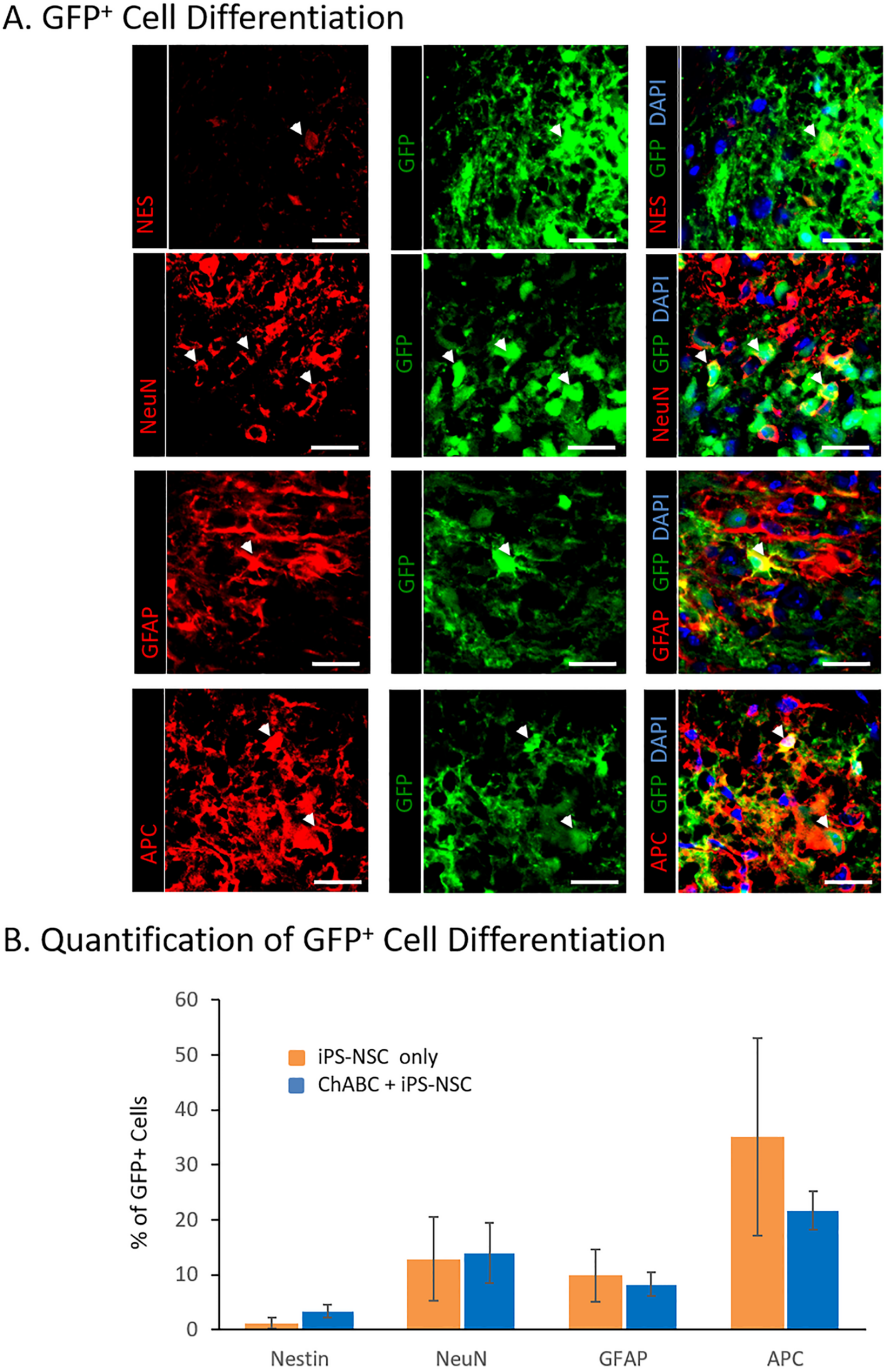
iPS-NSC differentiate along all three neuroglial lineages after transplant into ChABC pretreated chronically injured spinal cords. (A) Immunofluorescence imaging demonstrating undifferentiated neural stem cells (NES^+^/GFP^+^), neurons (NeuN^+^/GFP^+^), astrocytes (GFAP^+^/GFP^+^), and oligodendrocytes (APC^+^/GFP^+^) indicated by white arrowheads. Scale bar represents 50μm. (B) Quantification of GFP^+^ differentiated cell counts using optical fractionation and unbiased stereological techniques. iPS-NSC transplanted into aCSF (control) and ChABC pretreated cords demonstrated similar proportions of cell differentiation. Cell counting for immunofluorescent-positive cell shows a similar differentiation pattern for iPS-NSC with and without ChABC pretreatment. Data represent mean ± SEM, n = 6 per group.

Differentiation of transplanted GFP^+^ cells was assessed with immunostaining colocalization for markers of neural stem cells (Nestin^+^), mature neurons (NeuN^+^), motor neurons (NeuN^+^/ChAT^+^), oligodendrocytes (APC^+^) and astrocytes (GFAP^+^) (Fig 3A).

**Fig 4:**
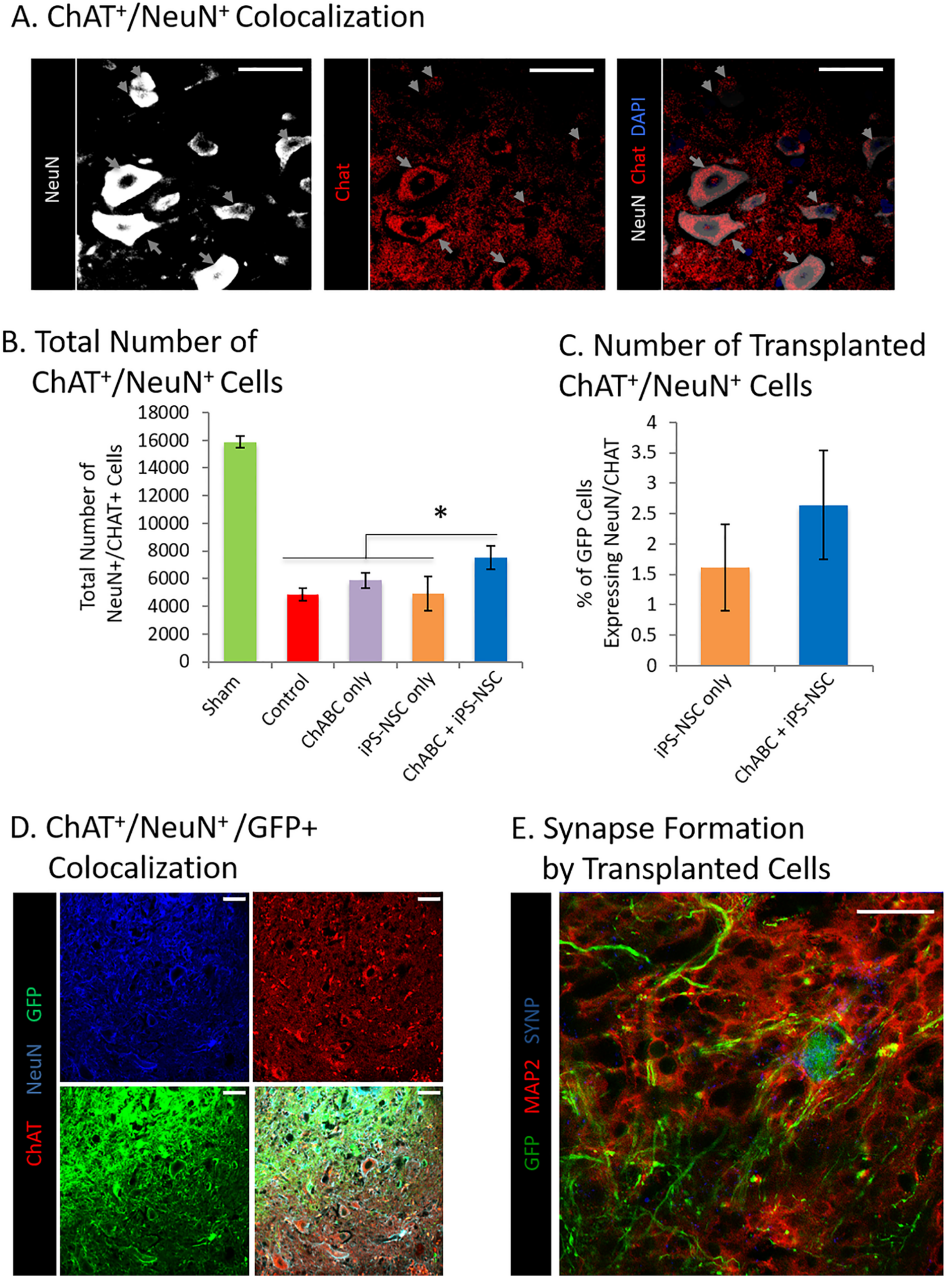
Combinatorial ChABC + iPS-NSC treatment results in preservation and regeneration of critically important acetylcholinergic neurons. (A) Representative images of ChAT^+^ and NeuN^+^ colocalization (gray arrowheads) in cells within the injured spinal cord gray matter of a ChABC + iPS-NSC treated animal. Scale bar 100 μm. (B) Quantification of NeuN^+^/ChAT^+^ cells. A significantly greater number of cells were found in the combinatorial treatment group than all other injury groups. (C) Proportion of transplanted GFP^+^ iPS-NSC differentiating to NeuN^+^/ChAT^+^ cells. There was no statistical difference in the proportion of GFP^+^/NeuN^+^/ChAT^+^ cells between transplanted groups, however, the absolute counts were much higher in the combinatorial group (390 vs 85 cells) due to the overall survival advantage with ChABC treatment. Data represent mean ± SEM, n = 6 per group. (*) indicates statistical significance at p<0.05. (D) Representative images of ChAT+/NeuN+/GFP+ and ChAT+/NeuN+/GFP- colocalization in cells within the injured spinal cord gray matter of a ChABC + iPS-NSC treated animal. Scale bar represents 100 μm. (E) Representative image of GFP+/MAP2+/Synaptophysin+ cells within the injured spinal cord gray matter of a ChABC + iPS-NSC treated animal. GFP+ cells are found to colocalize with the neuronal marker, MAP2, as well as the synapse marker providing evidence of synapse formation by transplanted cells. Scale bar 100 μm.

Higher absolute cell counts were found in the iPS-NSC + ChABC group compared to the iPS-NSC group for GFP^+^/Nestin^+^ (429 ± 123 vs 66 ± 45), GFP^+^/NeuN^+^ (2555 ± 863 vs 643 ± 246), GFP^+^/NeuN^+^/ChAT^+^ (390 ± 136 vs 85 ± 27), GFP^+^/APC^+^ (3407 ± 793 vs 1701 ± 711). However, proportional differentiation was not different between the groups for GFP^+^/Nestin^+^ (3.4 ± 1.2% vs 1.1 ± 1.0; p = 0.165), GFP^+^/NeuN^+^ (14.0 ± 5.5% vs 12.9 ± 7.6%; p = 0.914), GFP^+^/NeuN^+^/ChAT^+^ (2.6 ± 0.9% vs 1.6 ± 0.7%; p = 0.393), GFP^+^/APC^+^ (21.8 ± 3.4% vs 35.1 ± 17.9%; p = 0.484) or GFP^+^/GFAP^+^ (8.3 ± 2.2% vs 9.9 ± 4.8%; p = 0.765) (Fig 3B). Importantly, total numbers of acetylcholinergic neurons (ChAT^+^/NeuN^+^), a key population responsible for motor function, were significantly higher in the combined treatment group (Fig 4A, 4B, 4C and 4D).

In addition, numerous transplant-derived mature neurons (GFP^+^/MAP2^+^) formed synapses (Synaptophysin^+^) with host neurons in the iPS-NSC + ChABC group but not in the iPS-NSC alone group (Fig 4E). Furthermore, several of these transplant-derived neurons appeared to be myelinated with myelin basic protein^+^ (MBP^+^) cells (S3 Fig) highlighting the integration of transplanted cells and their ability to form appropriate nascent networks.

### ChABC treatment reduces astrogliotic and fibrous scar

**Fig 5:**
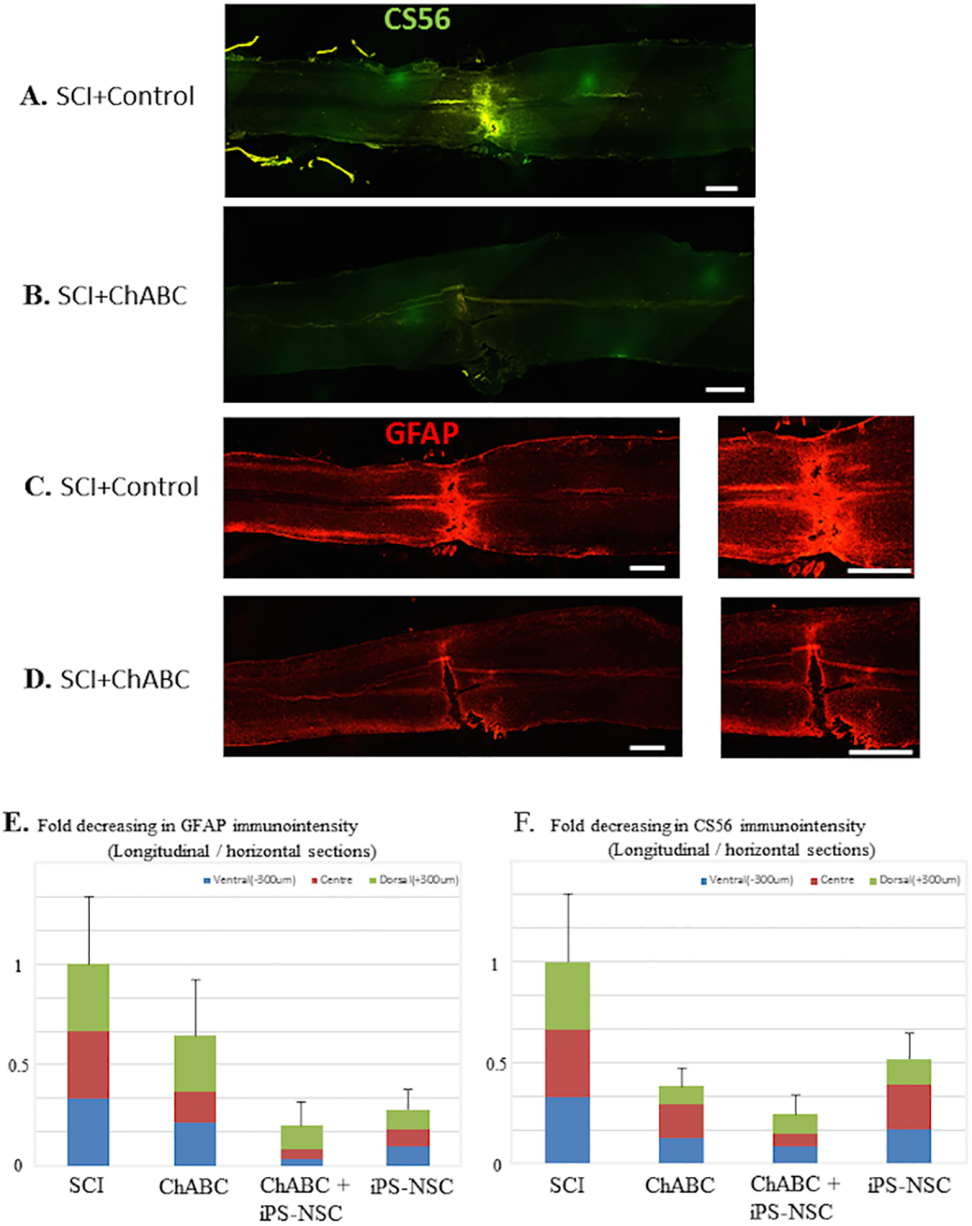
ChABC treatment reduces components of glial scar including astrocytes and chondroitin sulfate proteoglycans. Representative longitudinal and axial sections immunostained for CSPGs (CS56^+^) and astrocytes (GFAP^+^). (A, C) Extensive CSPG deposition and astrogliosis are found 16 weeks after injury in vehicle treated animals, however, (B, D) ChABC treatment 7 weeks after injury results in sustained reductions in CSPG and astrocyte labelling at 16 weeks post-injury. (E, F) Fold decreasing in GFAP and CS56 immunointensity in three longitudinal sections in each group (n = 2 per group). ChABC treated groups show a decrease in the total labelling area compared to the SCI group, however, statistical analysis was not performed because of the small number of animals. Scale bars = 500 μm (A-D).

Astrogliosis and fibrotic scar were assessed in longitudinal cord sections (Fig 5A, 5B, 5C and 5D). A dramatic reduction in CSPGs (CS56) and astrocytes (GFAP) can be seen in both the perilesional and lesional regions of the ChABC treated groups versus the control groups in keeping with numerous previous studies of ChABC for SCI. Quantification of fold decreasing in GFAP and CS56 immunointensity from 3 longitudinal slices in each group is provided in Fig 5E and 5F. The intensity values were normalized by dividing by measures in SCI group. These data were not analysed statistically due to an n of 2 per group.

### Transplanted iPS-NSCs form functional synapses

**Fig 6:**
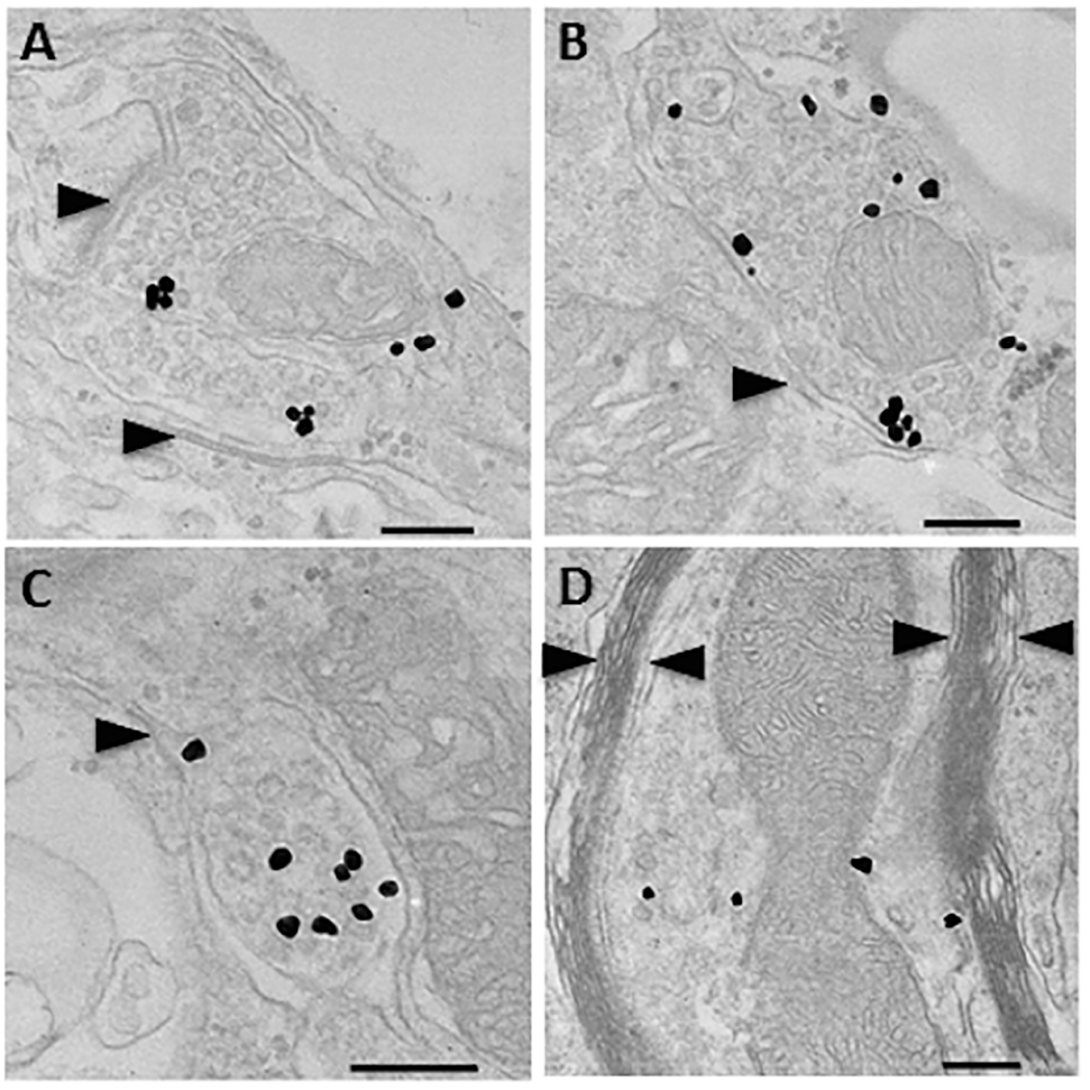
(A,B,C) Representative images of immune-transmission electron microscopy. Synapse formation (arrowheads) can be seen between host neurons and transplant-derived GFP+ (black labelled) neurons at 16 weeks post-injury. (D) Grafted GFP^+^ neuronal fibers were found in the injured spinal cord near the transplant site where they were myelinated by thick host myelin sheathes with more than 10 lamellae (between arrowheads). Scale bars = 200 nm (A-D).

Transmission electron microscopy confirmed that transplanted cells (immuno-labelled in black) formed synaptic connections with host neurons *in vivo* (Fig 6A, 6B and 6C). Furthermore, many graft-derived axons were densely myelinated by host cells with sheaths consisting of more than 10 lamellae (Fig 6D).

**Fig 7:**
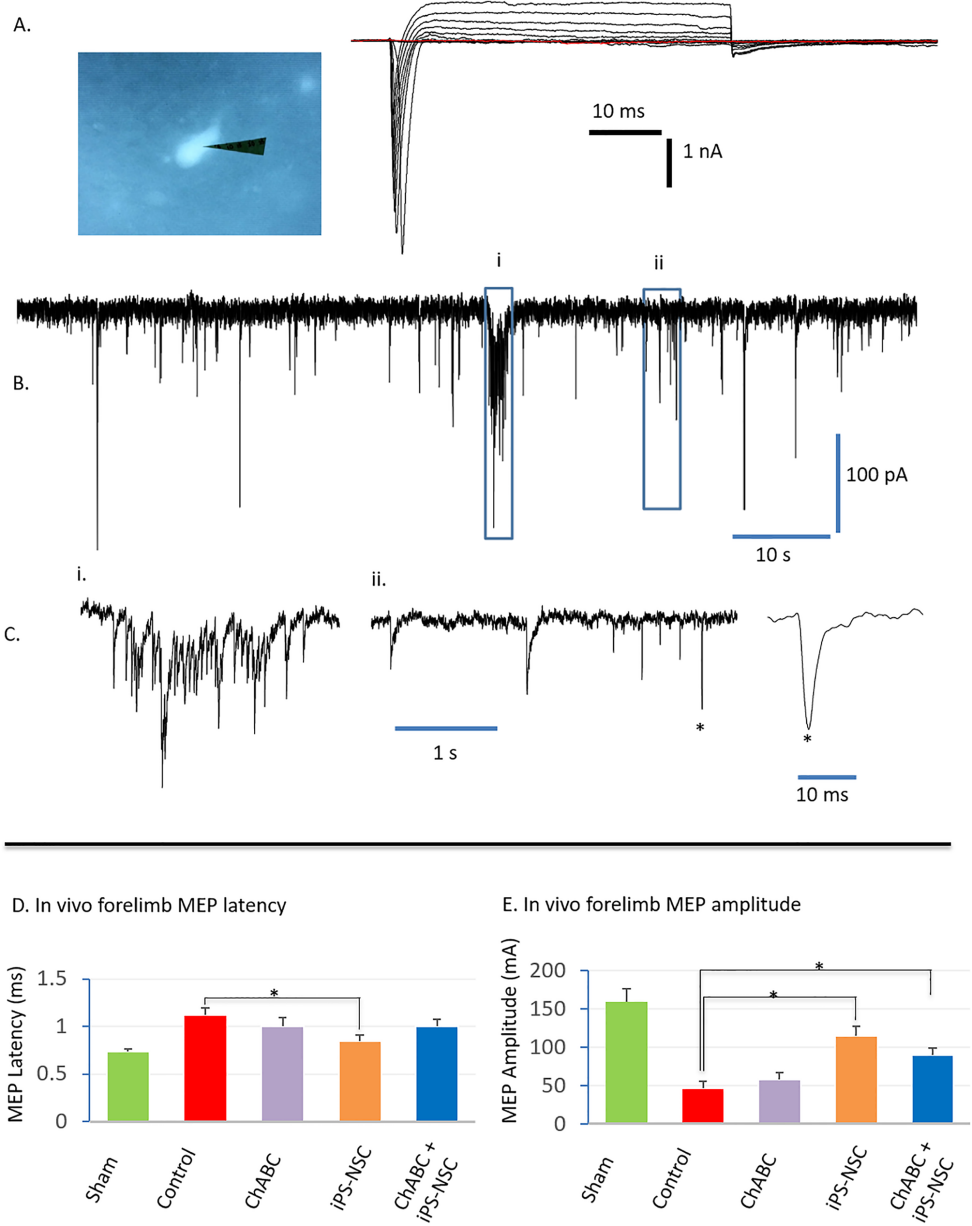
Patch-clamped transplanted GFP^+^ cells in *ex vivo* cords demonstrate functional action potentials. (A) Electrical tracing demonstrating currents evoked by voltage pulses which contain transient inward sodium currents and outward potassium currents. Membrane potential was held at -80 mV and depolarized to +30 mV with an increment of 10 mV step (48 ms long). (B) Representative spontaneous postsynaptic currents with burst and sporadic firing patterns. (C) Time expanded tracings of key (i) burst and (ii) sporadic postsynaptic currents in B. (ii) Two types of spontaneous postsynaptic currents are seen with slow decay time and (*) fast decay time. Membrane potential was held at -80 mV in B and C. n = 4 animals and 12 GFP^+^ cells per group. iPS-NSC transplant enhances recovery of in vivo motor evoked potentials (MEPs). MEPs were stimulated at the C2 cervical spinal cord and recorded from the hypothenar muscles. (D) At 16 weeks post-SCI, only mice treated with iPS-NSCs had significantly shorter MEP latencies while (E) higher peak MEP amplitudes were found in both the iPS-NSC and ChABC + iPS-NSC treated groups. Data are mean ± SEM values, n = 6 per group. (*) indicates statistical significance at p<0.05

At 8 weeks post-transplant, *ex vivo* whole cell patch clamp recording was performed on 24 of 42 GFP^+^ cells found in 8 mice (4 per iPS-NSC group). These cells were selected because they exhibited transient inward sodium currents of 0.6 nA or more (3.27 ± 0.35 nA) (Fig 7A) and spontaneous postsynaptic currents (Fig 7B). 20 of the selected cells demonstrated slow decay postsynaptic currents mediated by GABA_A_ receptors[33]. The other 4 cells contained both slow decay and fast decay postsynaptic currents, which were mediated by glutamatergic AMPA receptors (Fig 7C). Taken together, these results confirm that grafted iPS-NSC in chronic C6/7 injured spinal cords can differentiate into functional neurons capable of forming synapses within weeks of transplant [33].

### iPS-NSC transplant enhances *in vivo* electrophysiological outcomes

At 16 weeks post-injury, mice transplanted with iPS-NSC had significantly higher ventral forelimb muscle peak MEP amplitudes and shorter latency periods (higher conduction velocity) indicating a greater preservation and/or regeneration of motor tracts. Peak latency was measured at 0.73 ± 0.03ms in the sham group, 0.84 ± 0.07ms in the iPS-NSC group (p < 0.05), 1.0 ± 0.07ms in the iPS-NSC + ChABC group, 1.0 ± 0.09ms in the ChABC group and 1.12 ± 0.07ms in the untreated injury group (control)(Fig 7D). Peak amplitudes were measured at 159.4 ± 16.9mV in the sham group, 115.1 ± 12.8mV in the iPS-NSC group, 90.0 ± 8.7 in the combinatorial iPS-NSC + ChABC group, 58.0 ± 9.2mV in the ChABC group and 46.8 ± 9.2mV in the untreated injury group (Fig 7E). Only the iPS-NSC and combinatorial treatment groups had statistically significant peak amplitude improvements compared to untreated injured animals (p < 0.05).

### Combinatorial ChABC pre-treatment and iPS-NSC transplantation enhances behavioral recovery

There was no significant difference in grip strength, BMS, BMS motor subscore, stride length or swing speed between experimental groups during the pre-treatment period (week 1–6).

#### Forelimb grip strength

**Fig 8:**
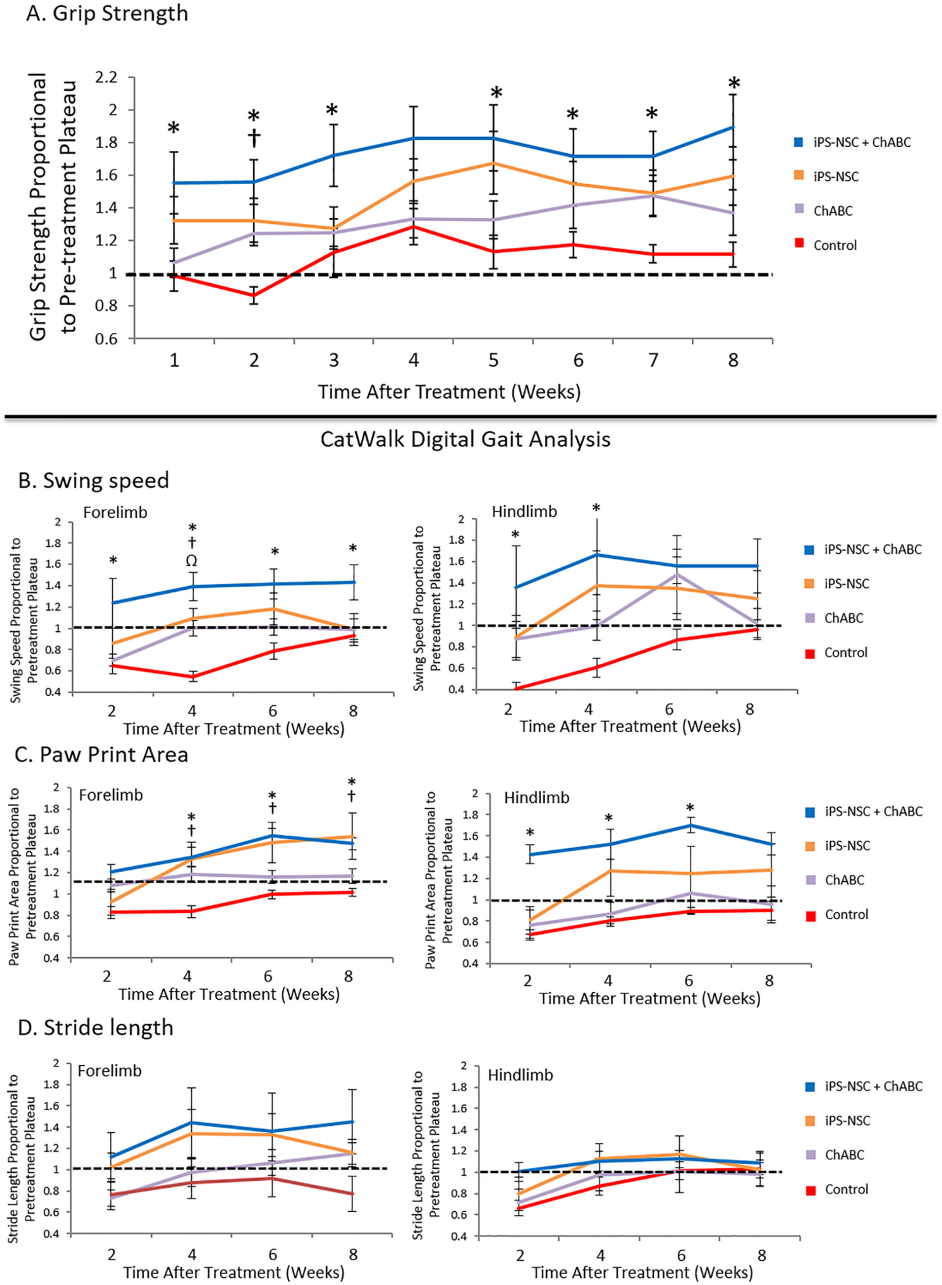
(A) iPS-NSC transplant combined with ChABC pretreatment improves recovery of forelimb grip strength in chronic SCI. Data are normalized to pretreatment (post-injury) baseline function with 1 representing no difference after treatment and values above 1 indicating improvement. The graph demonstrates a trend towards improvement (bar above 1) with ChABC or iPS-NSC treatment alone, however, only the combinatorial treatment with both resulted in statistically significant recovery across most post-treatment time points (p<0.05). Data represented as mean ± SEM. n = 15 per group. (*) indicates statistical significance for the iPS-NSC + ChABC group. (†) indicates significance for the iPS-NSC group. (B-D) Combined ChABC and iPS-NSC enhances CatWalk digital gait analysis outcomes. Data are normalized to pretreatment (post-injury) baseline function with 1 representing no difference after treatment and values above 1 indicating improvement. All comparisons are made against the control (injury alone) group. (B) Significant increases were seen in forelimb swing speed for the combinatorial treatment group only (p<0.05). Hindlimb swing speed improvements in the combinatorial group also reached statistical significance but only at the first two assessments (p<0.05). (C) Forelimb paw print area improved at the 4, 6, and 8-week time points in both the iPS-NSC and iPS-NSC + ChABC groups (p<0.05). Hindlimb paw print area only improved in the combinatorial group reaching statistical significance at the first three assessment time points (p<0.05). (D) The combinatorial group showed a nonsignficant trend towards improvement in forelimb stride length as well but not hindlimb stride length. Data represented as mean ± SEM n = 15 per group. (*) indicates statistical significance for the iPS-NSC + ChABC group. (†) indicates significance for the iPS-NSC group. (Ω) indicates significance for the ChABC group.

All animals demonstrated a gradual recovery of grip strength after injury which plateaued by 5 weeks. After treatment, grip strength differed between experimental groups (df = 3; F = 3.27; p = 0.03) with the ChABC and iPS-NSC groups demonstrating a nonsignificant trend towards improvement. The iPS-NSC group demonstrated a clear improvement compared to the control group, however, this did not reach statistical significance. Only the combined ChABC iPS-NSC group demonstrated a statistically significant improvement compared to the control group (μ difference = 8.68g [0.49–16.86g]; p = 0.034) (Fig 8A). At the conclusion of the study (16 weeks), grip strength was higher in the ChABC + iPS-NSC (39.4 ± 3.1g) and iPS-NSC (37.4 ± 3.0g) groups than the ChABC alone (29.7 ± 2.2g) or control (25.7 ± 1.9g) groups. This was further confirmed by standardizing post-treatment grip strength with each animal’s post-injury baseline function. While all groups demonstrated modest continued improvement post-treatment, only the combinatorial treatment resulted in significant recovery over most weeks compared to controls (p<0.05; Fig 8A).

#### Locomotor function

All experimental groups recovered gradually and plateaued at 3 weeks for BMS scores and 4 weeks for BMS subscores (S4 Fig). No significant difference was found between experimental groups after treatment (df = 3; F = 1.25; p = 0.30) or BMS subscores (df = 3; F = 0.60; p = 0.62).

Catwalk automated gait analysis was used to quantify the effects of treatment on a variety of gait parameters. Groups significantly differed on post-treatment measures of forelimb swing speed (df = 3; F = 3.89; p = 0.014) and hindlimb swing speed (df = 3; F = 2.82; p = 0.047). A clear trend did not emerge over time in the control, ChABC, or iPS-NSC groups, however, the combinatorial therapy group did demonstrate significant recovery on omnibus testing. This was confirmed by post hoc testing which found a statistically significant improvement in forelimb swing speed (μ difference = 0.081s [0.013–0.148]; p = 0.013) and hindlimb swing speed (μ difference = 0.126s [0.003–0.248]; p = 0.042) in the ChABC + iPS-NSC group compared to controls. At the conclusion of the study, forelimb swing speed was higher in the ChABC + iPS-NSC (0.309 ± 0.032s) group than the control (0.244 ± 0.021s), iPS-NSC (0.228 ± 0.016s), or ChABC (0.197 ± 0.025s) groups. Hindlimb swing speed was also higher in the ChABC + iPS-NSC (0.344 ± 0.049s) group than the control (0.273 ± 0.032s), iPS-NSC (0.264 ± 0.036s), or ChABC (0.219 ± 0.040s) groups (Fig 8B). When post-injury baseline function was used to standardize post-treatment scores, comparisons of swing speed remained the same and differences in paw print area were also seen. In particular, both the iPS-NSC and iPS-NSC + ChABC groups had significant improvements in forepaw print area at 4, 6, and 8 weeks post-treatment. Furthermore, hindpaw print area improved in the combinatorial treatment group to reach significance versus controls at 2, 4, and 6 weeks post-treatment (Fig 8C).

Forelimb stride length demonstrated a nonsignificant trend towards improvement in the combinatorial treatment group only (Fig 8D). No difference was seen between groups in hindlimb stride length.

## Discussion

Several groups, including our own, have demonstrated histopathological, electrophysiological and functional recovery after subacute transplantation of NSCs from different sources in animal models of subacute SCI[6, 34–36]. Unfortunately, the vast majority of patients are in the chronic phase of their injuries where the glial scar is well-established and has been shown to physically and biochemically interact with migrating cells and outgrowing axons to inhibit regeneration[37–39]. As a result, preclinical trials of cell therapy for chronic SCI have demonstrated varying degrees of success. A few studies have shown that exogenous cells can remyelinate denuded axons and preserve neural tissue in chronic SCI, however, electrophysiological and behavioral recovery has been limited[40–42]. Despite this, several key points have been learned from this work. First, grafted cells can survive in the chronic SCI niche but the survival rate is low[42]. Second, transplanted NSCs can differentiate to neurons but these neurons are severely limited in their ability to integrate with the host through functional synapses. Finally, when CSPGs are degraded, regeneration in the chronically injured cord can be enhanced[43]. Building on this data, we designed a combinatorial therapy aimed at improving transplanted NSC-mediated regeneration in the chronically injured cord by first modifying the local microenvironment using ChABC. Importantly, we did this in a clinically-relevant clip-contusion model of cervical SCI which closely mimics the pathophysiology of the human injury. Furthermore, we used the non-viral PiggyBac transposon system to generate translationally-relevant iPSC-derived NSCs. Finally, we focused on the recovery of upper limb function which is one of the most disabling losses after injury.

This study demonstrates a clear survival advantage for iPS-NSC grafted into ChABC pretreated animals (7.88 ± 1.60% vs 2.44 ± 1.04%) suggesting that the harsh chronic SCI niche can be ‘unlocked’ to be more conducive to regeneration. Importantly, while greater numbers of exogenous cells survived, the differentiation profile remained unchanged with progeny of all three neuroglial lineages being found throughout the epicenter and perilesional regions. Proportional differentiation was similar to a recently published study of this cell line by Salewski, et al. which used markers of oligodendrocytes (Olig2; 56.9 ± 0.4%), astrocytes (GFAP; 4.0 ± 0.5%), and neurons (NeuN; 16.0 ± 0.4%)[34]. Furthermore, there was no evidence of tumorigenicity in these non-virally derived cells with or without ChABC treatment as demonstrated by the graft morphology and a very low Ki67^+^/GFP^+^ colocalization rate in both groups. An extended experimental period beyond 8 weeks post-transplant will be required to definitively confirm safety.

The integration of our transplanted cells into chronically injured, ChABC treated cords parallels findings from prior studies of NSC transplants in chronic SCI[35, 41]. Those studies demonstrated remyelination of denuded host axons by grafted cells as well as myelination of transplant-derived neurons. We similarly found host and graft cells to be closely associated along long myelinated fibers *in vivo* (S3 Fig). Immunoelectron microscopy confirmed that graft-derived neuronal fibers were being myelinated by thick host myelin sheaths (Fig 6D). Moreover, transplant-derived neurons were found to express synaptic vesicle protein, synaptophysin (Fig 4E), and showed clear synapse formation on immuno-TEM with host cells (Fig 6A, 6B and 6C). Secondly, greater numbers of endogenous and exogenous acetylcholinergic motor neurons were found in ChABC treated cords representing a key population of cells capable of reforming neural circuits to enhance recovery. *Ex vivo* patch clamp examination revealed that transplanted cells were forming functional acetylcholinergic and glutamatergic neurons with spontaneous burst and delayed firing patterns. This provides strong evidence that iPS-NSC-derived neurons can integrate into complex local circuits with both inhibitory and excitatory components.

The *in vivo* electrophysiologic and behavioral data demonstrated recovery reaching levels more often seen in subacute cervical injury[29, 44]. Measures of long tract conductance to the functionally-important forelimbs found that cell transplant with or without ChABC resulted in enhanced conduction amplitude similar to Wilcox et al[19]. This was reflected in improvements on grip strength testing for the combinatorial treatment group across all post-treatment time points. Interestingly, while MEP conduction velocity and amplitude improved more in the iPS-NSC group than the combinatorial treatment group, the combinatorial treatment resulted in a significantly better behavioral outcome. The disconnect between these two measures has been previously observed in animal models and may be due to recovery levels below the detection threshold of currently-available gross behavioral tests[45, 46]. An additional key measure, the CatWalk digital gait analysis, can be used to quantify changes in locomotion that result from SCI and the subsequent recovery[28, 47]. We found that combinatorial treatment resulted in the greatest improvements in fore-/hindlimb swing speed and paw print area. The Catwalk data presented here corresponds with previous work showing that NSC transplant can promote recovery of swing speed in a mouse model of thoracic injury[41]. An initial decrease in swing speed, stride length, and print area was observed, particularly in the control and single-treatment groups, compared with their pre-treatment plateau. This decrease was likely due to the morbidity of the multi-injection intraparenchymal transplant procedure. This effect was may be masked by the magnitude of recovery in the combinatorial group. BMS and BMS subscores did not reveal any difference between groups. This is likely because this test is designed to measure deficits in a thoracic model of injury and is not sensitive enough to detect behavioral changes in our cervical injury model. In fact, previous work indicates that BMS scoring may not be as reliable as Catwalk due to the subjective nature of the scoring process[48]. Overall, the results suggest that both motor and sensory function can be improved using this unique combinatorial strategy. Importantly, additional work is required to pinpoint the exact neuroanatomical mechanisms behind this recovery. Given the time course, it is likely that transplant-derived trophic support and ChABC-induced ECM changes aid in the survival/regeneration of endogenous cells[49], even in the chronic phase of injury[50]. The extent to which each of these mechanisms contributes to the overall recovery profile requires further investigation.

The main limitations of our work are: (1) the duration of the post-treatment experimental period could be extended to assess the definitive long-term integration of grafted cells into the host cord, (2) somatosensory evoked potential (SSEP) testing would have been valuable to add corroborating evidence of sensory recovery, and (3) these findings are applicable to the more common cervical injury niche only and additional studies will be required to validate this strategy for thoracic injury. The next steps will be to adapt this treatment protocol to translationally-relevant human cell lines while exploring longer experimental timelines to assess safety and graft integration.

## Conclusion

We report a novel therapeutic strategy using intrathecal ChABC pretreatment to ‘unlock’ the chronically injured spinal cord microenvironment and enhance subsequent cell-based therapy. We demonstrate a significant graft survival advantage with this technique and show clear evidence of behavioral recovery in a clinically-relevant model of SCI. These findings have important clinical implications for the vast majority of patients who are in the chronic phase of their injuries where even small improvements in hand motor function can have tremendous impacts on their quality of life.

## Supporting information

S1 Fig A cervical SCI model at level C6/7 and ChABC injection with a mini-osmotic pump. (A) A modified aneurism clip. (B) Injury site after spinal cord injury at C6/7. (C) (E) At 6 weeks after SCI and 1 week prior to cell transplantation, mice had a mini osmotic pump surgically implanted containing either artificial cerebrospinal fluid (aCSF) or aCSF with ChABC. Treatments were administered intrathecally using a fine catheter connected to an osmotic mini-pump (Alzet pump model No.1007D, 0.5 ml/hr.) for 7 days as we reported previously. (D) A fine catheter (Alzet, mouse IT, 0007743, 0.23 mm OD; 0.09 mm ID).
(TIF)

S2 Fig Transplanted GFP^+^ iPS-NSCs demonstrate no evidence of proliferation at 8 weeks post-transplant. Ki67, a marker of cell proliferation was not found to colocalize with GFP^+^ cells in any group. Scale bar represents 50μm. n = 6 per iPS-NSC transplanted group.
(TIF)

S3 Fig Evidence of host-graft integration with colocalization of myelin (MBP^+^), neurons (NF200^+^) and GFP^+^ cells along a nearly linear path (gray arrowheads). Whether exogenous cells are remyelinating endogenous cells or vice versa could not be determined, however, endogenous (GFP^-^) and exogenous (GFP^+^) cells are integrating to form myelinated axons. Scale bars represent 50μm (left panels) and 25 μm (right panels).
(TIF)

S4 Fig Basso mouse scale (BMS) analysis for locomotor function following SCI. Weekly open field analysis and quantification using the (A) traditional 9-point BMS scale or (B) the BMS motor sub-score did not find any significant differences between the control and treatment groups. A plateau was found in all group at approximately 5 for BMS and 4 for the BMS sub-score. Data represented as mean ± SEM.
(TIF)

S1 Table Specific treatments within each control and experimental group. (DOCX)

S2 Table Antibodies. (DOCX)

S3 Table Summary of double and triple immunohistochemical cell quantifications. (DOCX)
