# Supplementary figures and images for: Neural stem cell mediated recovery is enhanced by Chondroitinase ABC pretreatment in chronic cervical spinal cord injury
Source: PLoS One. 2017 Aug 3;12(8):e0182339. doi: 10.1371/journal.pone.0182339 (PMC5542671; doi:10.1371/journal.pone.0182339)

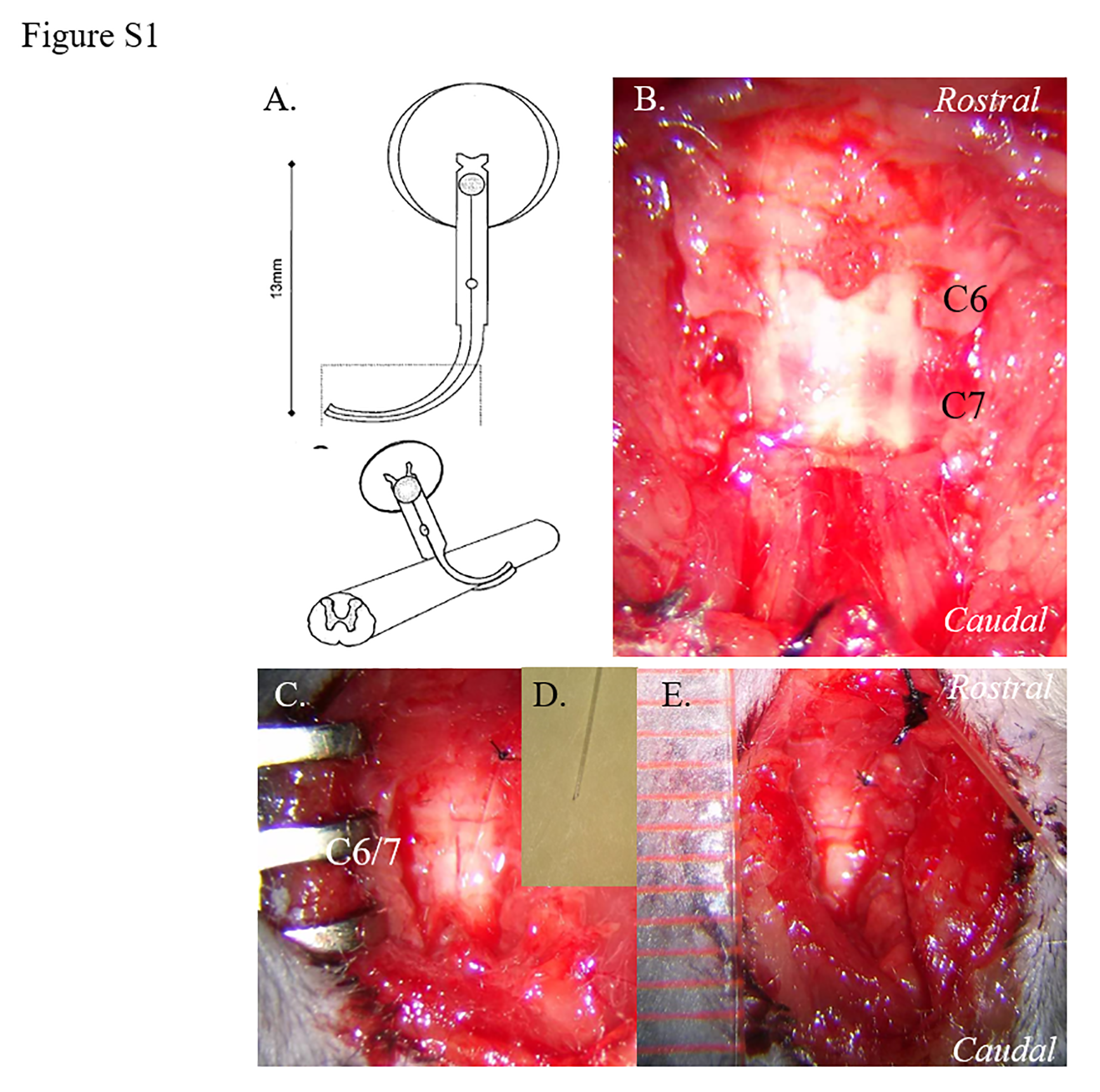

Supplement: S1 Fig — (A) A modified aneurism clip. (B) Injury site after spinal cord injury at C6/7. (C) (E) At 6 weeks after SCI and 1 week prior to cell transplantation, mice had a mini osmotic pump surgically implanted containing either artificial cerebrospinal fluid (aCSF) or aCSF with ChABC. Treatments were administered intrathecally using a fine catheter connected to an osmotic mini-pump (Alzet pump model No.1007D, 0.5 ml/hr.) for 7 days as we reported previously. (D) A fine catheter (Alzet, mouse IT, 0007743, 0.23 mm OD; 0.09 mm ID). (TIF) [file pone.0182339.s001.tif]

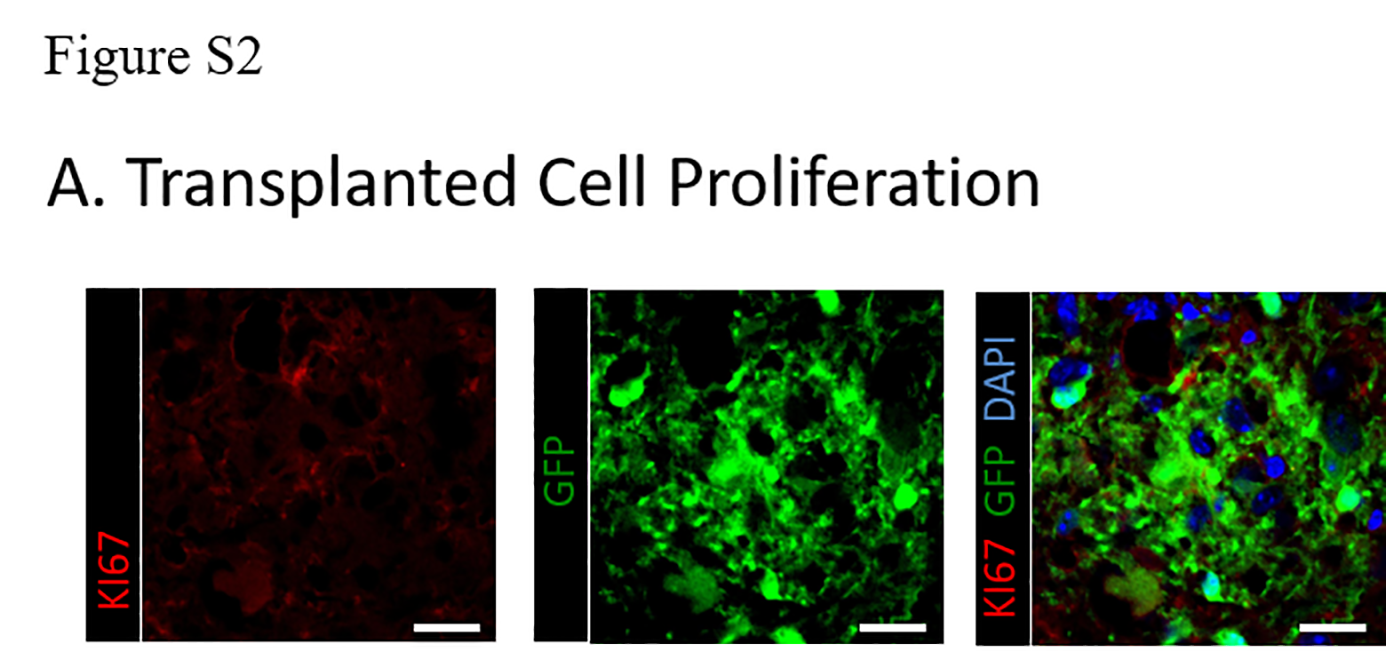

Supplement: S2 Fig — Ki67, a marker of cell proliferation was not found to colocalize with GFP+ cells in any group. Scale bar represents 50μm. n = 6 per iPS-NSC transplanted group. (TIF) [file pone.0182339.s002.tif]

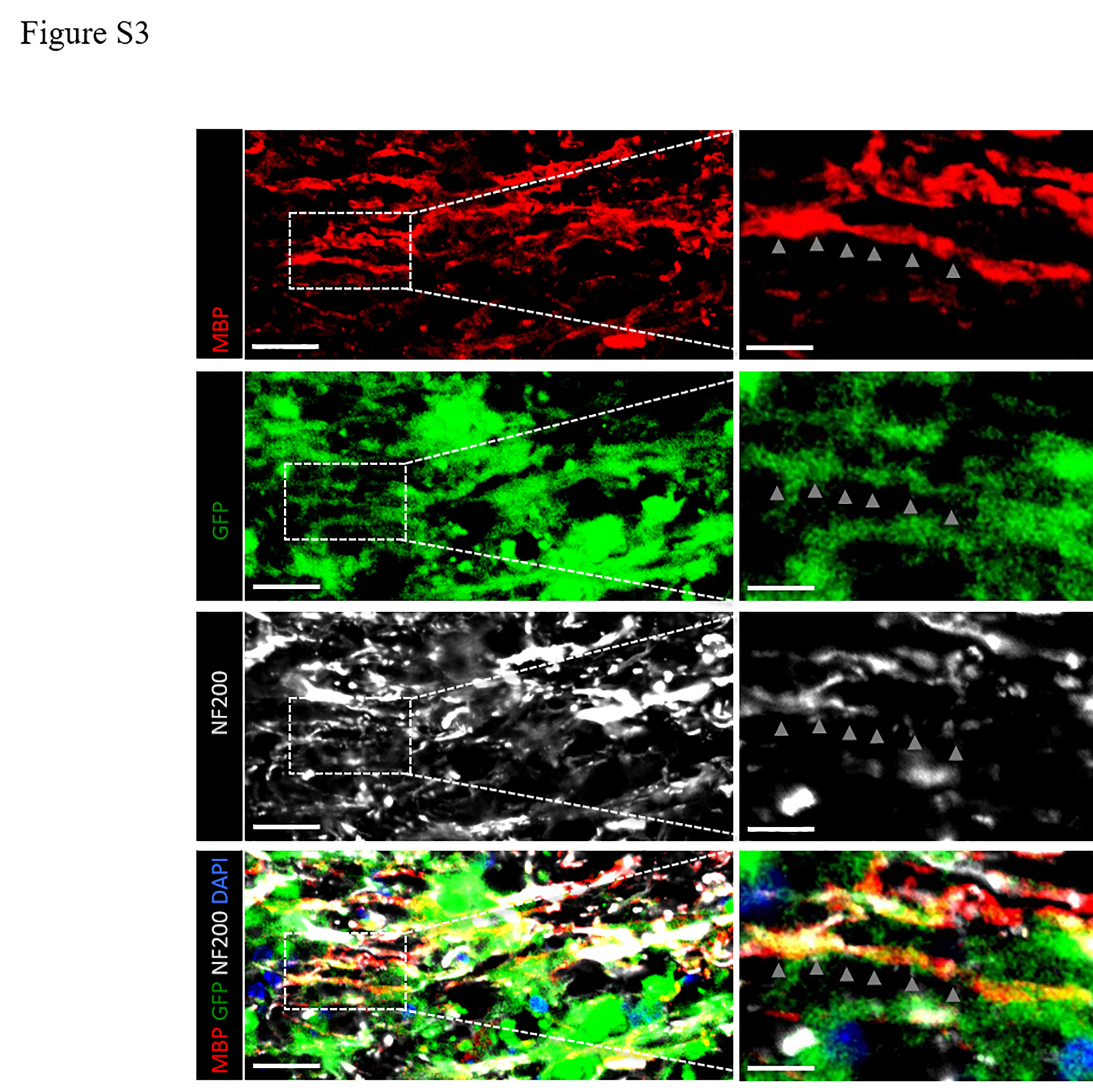

Supplement: S3 Fig — Whether exogenous cells are remyelinating endogenous cells or vice versa could not be determined, however, endogenous (GFP-) and exogenous (GFP+) cells are integrating to form myelinated axons. Scale bars represent 50μm (left panels) and 25 μm (right panels). (TIF) [file pone.0182339.s003.tif]

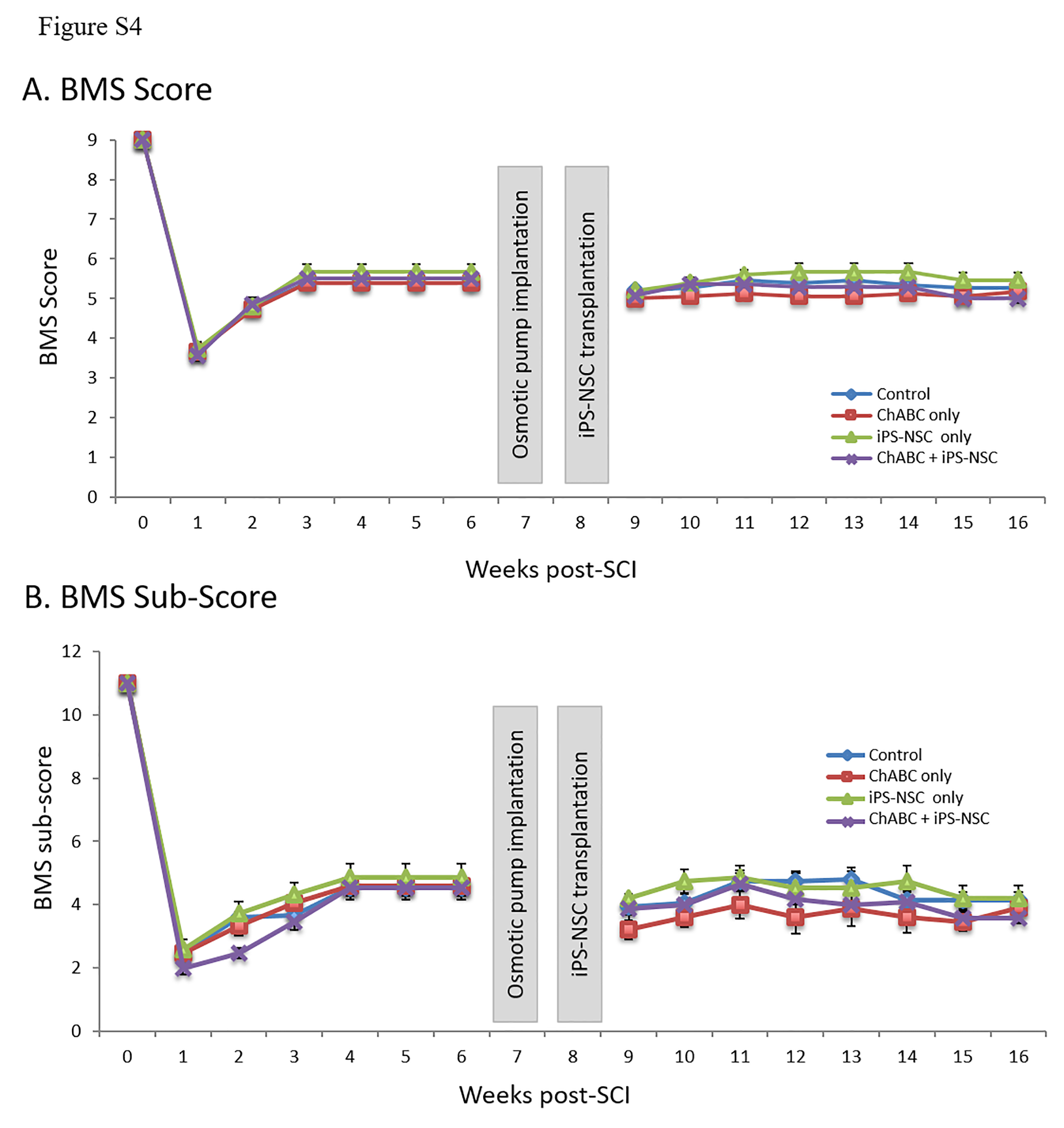

Supplement: S4 Fig — Weekly open field analysis and quantification using the (A) traditional 9-point BMS scale or (B) the BMS motor sub-score did not find any significant differences between the control and treatment groups. A plateau was found in all group at approximately 5 for BMS and 4 for the BMS sub-score. Data represented as mean ± SEM. (TIF) [file pone.0182339.s004.tif]
