# Supplementary material for: Neural stem cell mediated recovery is enhanced by Chondroitinase ABC pretreatment in chronic cervical spinal cord injury
Source: PLoS One. 2017 Aug 3;12(8):e0182339. doi: 10.1371/journal.pone.0182339 (PMC5542671; doi:10.1371/journal.pone.0182339)
Supplement: S1 Table — (DOCX) [file pone.0182339.s005.docx]

|  | C6/7 Laminectomy | Spinal Cord Injury | aCSF | ChABC | iPS-  NSC | PBS | Mino | CsA | n | Mortality |
| --- | --- | --- | --- | --- | --- | --- | --- | --- | --- | --- |
| Sham | ＋ | － | － | － | － | － | － | － | 15 | 0 |
| Control | ＋ | ＋ | ＋ | **－** | **－** | ＋ | ＋ | ＋ | 16 | 1 |
| ChABC | ＋ | ＋ | － | **＋** | **－** | ＋ | ＋ | ＋ | 16 | 1 |
| iPS-NSC | ＋ | ＋ | ＋ | － | **＋** | － | ＋ | ＋ | 16 | 2 |
| ChABC + iPS-NSC | ＋ | ＋ | － | **＋** | **＋** | － | ＋ | ＋ | 17 | 2 |

S1 Table. Specific treatments within each control and experimental group.

Abbreviations: aCSF - artificial cerebrospinal fluid; ChABC - chondroitinase ABC; CsA - Cyclosporine A; iPS-NSC – induced pluripotent stem cell derived neural stem cells; Mino – minocycline; PBS – phosphate buffered saline
