# Supplementary material for: Neural stem cell mediated recovery is enhanced by Chondroitinase ABC pretreatment in chronic cervical spinal cord injury
Source: PLoS One. 2017 Aug 3;12(8):e0182339. doi: 10.1371/journal.pone.0182339 (PMC5542671; doi:10.1371/journal.pone.0182339)
Supplement: S2 Table — (DOCX) [file pone.0182339.s006.docx]

S2 Table. Antibodies.

| Antibody | Specificity | Source | Dilution factor |
| --- | --- | --- | --- |
| Nestin | Neural stem/progenitor cells | Chemicon, MAB353 | 1:100 |
| GFAP | Astrocytes | Chemicon, MAB3402 | 1:1000 |
| CS56 | CSPGs | Sigma, C8035 | 1:200 |
| APC | Mature oligodendrocytes | Calbiochem, OP80 | 1:50 |
| NeuN | Mature Neurons | Chemicon, MAB377 | 1:500 |
| MAP2 | Mature Neurons | Abcam, ab5392 | 1:200 |
| MBP | Myelin | Santa Cruz, sc-13914 | 1:500 |
| NF200 | Axons | Sigma, N0142 | 1:500 |
| Synaptophysin | Synaptic boutons | Chemicon, MAB5258 | 1:50 |
| ChAT | Motor neurons | Millipore, AB144 | 1:100 |
| Ki67 | Cell proliferation | Chemicon, MAB4190 | 1:300 |
| Alexa 647, 568,  488 and 350 | Secondary antibody | Invitrogen | 1:400 |
