# Supplementary material for: Neural stem cell mediated recovery is enhanced by Chondroitinase ABC pretreatment in chronic cervical spinal cord injury
Source: PLoS One. 2017 Aug 3;12(8):e0182339. doi: 10.1371/journal.pone.0182339 (PMC5542671; doi:10.1371/journal.pone.0182339)
Supplement: S3 Table — (DOCX) [file pone.0182339.s007.docx]

S3 Table. Summary of double and triple immunohistochemical cell quantifications.

| Antibody | Cell type and criteria for cell counting |
| --- | --- |
| Nestin / GFP / DAPI | Nestin + / GFP + /DAPI +  : Neural progenitor cells differentiated from IPS-NSCs |
| NeuN / ChAT / GFP / DAPI | NeuN + / ChAT + / GFP - / DAPI +  : Endogenous neurons  NeuN + / ChAT - / GFP - / DAPI +  : Endogenous cholinergic neurons  NeuN + / ChAT + / GFP + / DAPI +  : Mature neurons differentiated from IPS-NSCs  NeuN + / ChAT - / GFP + / DAPI +  : Mature cholinergic neurons differentiated from IPS-NSCs |
| APC / GFP / DAPI | APC + / GFP + / DAPI +  : Mature oligodendrocytes differentiated from IPS-NSCs |
| Ki67 / GFP/ DAPI | Ki67 - / GFP + / DAPI +  : Nonproliferation cells from IPS-NSCs  Ki67 + / GFP + / DAPI +  : Proliferating cells from IPS-NSCs |
| MAP2 / Synaptophysin / GFP / DAPI | MAP2 + / Synaptophysin + / GFP + / DAPI +  : Neurons with synaptic formation differentiated from IPS-NSCs |
| MBP / NF200 / GFP / DAPI  (longitudinal section) | MBP + / NF200 +  : Entirely endogenous cell derived myelinated axons  MBP + / NF200 + / GFP + / DAPI +  : Myelinated axons at least partially derived from IPS-NSCs |
| GFAP / CS56  (longitudinal section) | GFAP + / CS 56 - : Astrogliosis area  GFAP - / CS56 + : CSPG area |
